# Recent advances in understanding Antarctic subglacial lakes and hydrology

**DOI:** 10.1098/rsta.2014.0306

**Published:** 2016-01-28

**Authors:** Martin J. Siegert, Neil Ross, Anne M. Le Brocq

**Affiliations:** 1 Grantham Institute and Department of Earth Science and Engineering, Imperial College London, South Kensington, London SW7 2AZ, UK; 2 School of Geography, Politics and Sociology, Newcastle University, Claremont Road, Newcastle Upon Tyne NE1 7RU, UK; 3 Geography, College of Life and Environmental Sciences, University of Exeter, Exeter EX4 4RJ, UK

**Keywords:** ice sheet, basal processes, ice flow, Antarctica

## Abstract

It is now well documented that over 400 subglacial lakes exist across the bed of the Antarctic Ice Sheet. They comprise a variety of sizes and volumes (from the approx. 250 km long Lake Vostok to bodies of water less than 1 km in length), relate to a number of discrete topographic settings (from those contained within valleys to lakes that reside in broad flat terrain) and exhibit a range of dynamic behaviours (from ‘active’ lakes that periodically outburst some or all of their water to those isolated hydrologically for millions of years). Here we critique recent advances in our understanding of subglacial lakes, in particular since the last inventory in 2012. We show that within 3 years our knowledge of the hydrological processes at the ice-sheet base has advanced considerably. We describe evidence for further ‘active’ subglacial lakes, based on satellite observation of ice-surface changes, and discuss why detection of many ‘active’ lakes is not resolved in traditional radio-echo sounding methods. We go on to review evidence for large-scale subglacial water flow in Antarctica, including the discovery of ancient channels developed by former hydrological processes. We end by predicting areas where future discoveries may be possible, including the detection, measurement and significance of groundwater (i.e. water held beneath the ice-bed interface).

## 1. Introduction and background

Antarctic subglacial lakes were first detected using radio-echo sounding (RES) in the late 1960s [1], from a geophysical survey of the Antarctic Ice Sheet that took place between 1968 and 1979 [2]. Subglacial lakes are easily identifiable in RES data, as the bright, flat specular reflections from an ice–water interface are distinct from the rough, variable power reflections from bedrock or sediment. The first inventory of 17 lakes [3] revealed that the centre of the East Antarctic Ice Sheet supported a widespread collection of subglacial water bodies. This was followed by further discoveries, including the first detection of Lake Vostok, the largest Antarctic subglacial lake [4]. Subglacial lakes exist because ice is a particularly good thermal insulator. With only background levels of geothermal heating, if the ice is sufficiently thick, the temperature at the bed can reach the pressure melting value. Subglacial water will flow under gravity and ice overburden pressure and pool where the hydrological potential is at a minimum. Such minima can form within discrete topographic basins and over flatter topography as a consequence of a localized shallow ice thickness gradient resulting from ice flow variations. As ice-surface slopes have an order of magnitude greater influence on water flow than basal slopes [5] a phenomenon can occur in which basal water flows uphill provided basal slopes are less than 10 times (and in the opposite direction to) the ice surface.

Subsequent to the discovery of subglacial lakes over 40 years ago, the consensus among glaciologists at the time was that water flowed very slowly at the Antarctic Ice Sheet bed and, therefore, had minimal glacial dynamical impact. As a consequence, little research on subglacial lakes was conducted in the 1980s, except for an unpublished chapter in a PhD thesis [6]. This lack of research coincided with the cessation of large-scale long-range RES sounding [7,8], in favour of smaller scale hypothesis-driven data acquisition.

Interest in subglacial lakes was renewed in the early 1990s, when high-precision ERS-1 satellite radar altimetry revealed that the 1970s RES data from Lake Vostok coincided with a notably flat ice surface [9]; the flat surface being caused by the change in ice dynamics from grounded ice shearing to lateral extension when afloat. The association between the two datasets implied that the lake was more than 200 km long and approximately 50 km wide. Through a remarkable episode of serendipity, the satellite data also revealed that Vostok Station, the site of the deep palaeoclimate ice core, was located at the southern extreme margin of the lake. Seismic investigations at Vostok Station had been conducted in the early 1960s by Russian scientists to determine the ice thickness, so it was possible that these old data contained a record of the lake floor. A meeting was convened in Cambridge, UK, in 1994, with the (now retired) scientists responsible for the data acquisition and those familiar with the satellite observations, to investigate what the multiple datasets collectively revealed about the lake. Thus, the first multi-national collaborative work on Lake Vostok was formed leading to the discovery that it is at least 500 m deep [10]. This breakthrough paper—the first to measure the water depth of a subglacial lake—led to awareness that this and other lakes were extreme yet viable habitats for microbial life and holders of ancient climate records [11].

In the 20 years that followed, our appreciation of Antarctic subglacial lakes developed considerably and allowed a wider appreciation of hydrological processes beneath the ice. We now know that over 400 lakes exist at the ice-sheet bed. The last full inventory of subglacial lakes provided information on 379 features [12]; this was updated later in the same year with the publication of a further 19 lakes across Dome C and the Aurora Subglacial Basin [13] and more recently by the discovery of three lakes in the upper catchment of Byrd Glacier [14] and one at the West Antarctic ice divide [15], taking the tally of published Antarctic subglacial lakes to 402, with several additional lakes noted in as yet unpublished records.

Some subglacial lakes are prone to sudden discharges of water (‘active’ subglacial lakes), which can flow hundreds of kilometres and also connect with other lakes [16]. Such water flow can influence the short-term flow of ice above [17] and many lakes are associated with the onset of enhanced flow acting as potential regulators of ice-sheet dynamics [18,19]. We also know that Greenland contains a low number (currently four) of subglacial lakes [20,21,22] towards the edges of the ice sheet.

The last review on the state of knowledge of subglacial lakes and their impact on ice-sheet hydrology was undertaken by Wright & Siegert [23]. An excellent recent review of subglacial hydrology is provided in Ashmore & Bingham [24]. Here, we concentrate on discoveries since 2011 that further add to our view of Antarctic subglacial lakes as a diverse, dynamic and intriguing system, on which further exciting research awaits.

## 2. Satellite altimetric detection of active subglacial lakes

Satellite altimetry techniques have been used on numerous occasions to identify the presence of ‘active’ subglacial lakes, their connectivity and their impact on ice dynamics [16,17,25,26]. The assumption of these studies is that any short-term focused variation in the surface elevation of the ice sheet is an expression of the movement of water at the bed (i.e. the draining or filling of subglacial water bodies). Along-track measurements of ice-surface elevation using the ICESat satellite, operational between 2003 and 2009, were used to produce the first continent-scale map of ‘active’ subglacial lakes in Antarctica [27]. The distribution of ‘active’ subglacial lakes is distinct from those within the deep continental interior [12], as the majority of ‘active’ lakes are located between ice-stream-onset zones and the ice-sheet margin [28].

One of the most remarkable ‘active’ subglacial lakes identified by Smith *et al*. [27] is Lake Cook, located in Victoria Land, East Antarctica. A suite of remote sensing methods have recently been used to monitor and investigate this subglacial lake, including ICESat, CryoSat-2 [29], and ASTER and SPOT5 satellite imagery [30]. The data show that the drainage of Lake Cook led to drawdown of the ice-sheet surface of approximately 70 m between 2007 and 2008 and the formation of a 260 km^2^ surface depression. Between 2009 and 2012, the lake re-filled slowly, causing the ice-sheet surface to slowly rise again [29,30]. The Lake Cook outburst represents the largest single subglacial drainage event (5.2±1.5 km^3^) yet reported from Antarctica [30]. Analysis of radar altimetry down-ice of the lake demonstrated the impact its drainage had on downstream lakes, providing evidence for a 500 km long connected hydrological system in the Wilkes Basin [30].

Since the publication of Smith *et al*. [27], several RES surveys have targeted ‘active’ subglacial lakes (e.g. [14,31,32,33]). A significant proportion of these have found little or no evidence for the presence of significant volumes of subglacial water beneath the observed ice-surface anomalies. This may be for several reasons: (i) the change in ice-surface elevation was not caused by the movement of subglacial water (i.e. the measurements do not represent ‘lakes’); (ii) the lakes were empty at the time of the RES survey; (iii) the central coordinates of the mapped surface area of the ‘active’ lakes targeted by the RES surveys are offset from the locality which experienced the greatest range of surface elevation change observed by satellite altimetry (i.e. the subglacial water body was more limited than the area identified and reported by Smith *et al*. [27] and the RES surveys did not target the correct locality); or (iv) the surface elevation expressions are artefacts of the ICESat data and their analysis. The latter is most likely to occur when the surface elevation change is negligible (i.e. within or close to the uncertainties associated with the data), or when the ‘active’ lakes were inferred from a small number of ICESat tracks (i.e. one or two passes).

**Figure 1.**
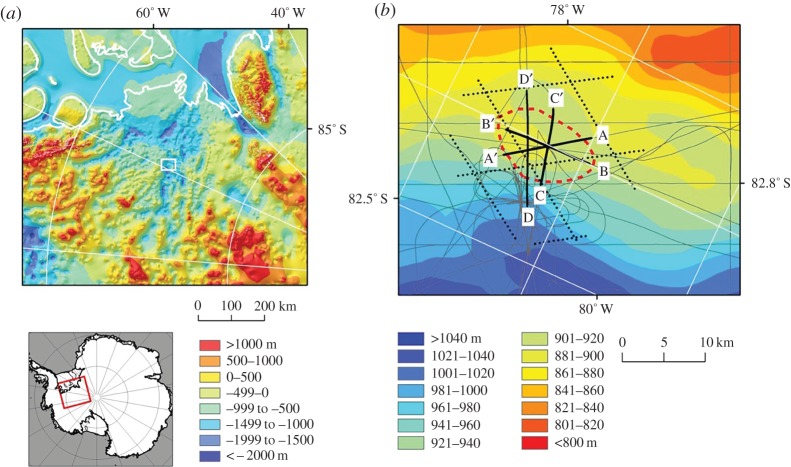
Geophysical investigations of ‘active’ subglacial lake Institute E2. (*a*) Subglacial topography of the Institute Ice Stream region (with insert for the study region in West Antarctica) [34]. The grounding line is provided in white. Elevations are in metres above WGS84. (*b*) Ice-surface elevation with RES (grey lines) [35] and ICESat transects (black dotted lines) over and around Institute E2 (dashed line), as delineated by Smith *et al*. [27]. RES lines in black and labelled A–A’, B–B’ and C–C’ refer to RES transects provided in figure 2. Elevations are in metres above WGS84. (Adapted from Siegert *et al*. [33].)

**Figure 2.**
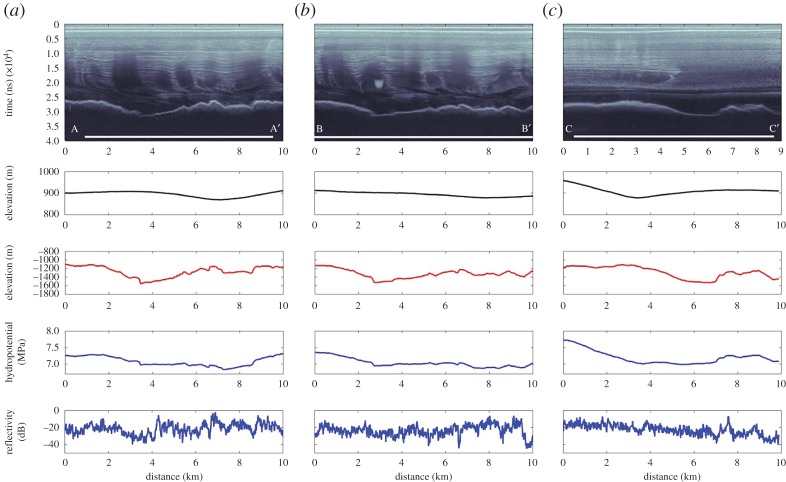
RES transects centred on Institute E2. The locations of the transects are provided in figure 1*b*. (*a*) Transect A–A’, (*b*) transect B–B’ and (*c*) transect C–C’. For each transect, the coverage of the ICESat-derived lake extent (after [27]) is shown as a white bar on the radargram. Beneath the radargrams graphs of ice-surface elevation (m WGS84), bed elevation (m WGS84), basal hydropotential and basal reflectivity are provided. (Adapted from Siegert *et al*. [33].)

To investigate the nature of the subglacial environment directly beneath and around a zone of known ice-surface elevation change, Siegert *et al*. [33] undertook a targeted airborne survey of the ‘active’ subglacial lake named Institute E2, located in the upper catchment of the Institute Ice Stream in West Antarctica. The survey included a polarimetric set of profiles, centred on the published coordinate for the middle of the ‘active’ lake [27], as well as numerous transects surrounding the region. The result was a high-resolution image of the ice-sheet topography around the ‘active’ lake and radio-wave reflection data collected at a variety of orientations over the lake (figure 1). Two main observations were made from the data, which help us understand the nature of Institute E2.

The first thing to note about the resulting dataset is that no obvious RES reflection from a deep-water subglacial lake was identified (figure 2). Normally, one might speculate that this could be because of the orientation of the profile (as subglacial corrugation is possible over subglacial lakes, which would lead to scattering of radio-wave reflections). However, as a polarimetric survey was conducted, this possibility can effectively be ruled out. Reflected power is also a good indication of subglacial water, as reflected powers from an ice–water interface are generally 10–20 dB greater than those from ice and rock [3]. The data for Institute E2 show a high degree of variability in reflected power, with some level of spatial coherence, but not consistent with the expected outline of the ‘active’ lake from surface observations (figures 1 and 2). The first conclusion from analysis of RES data is that there is no evidence for Institute E2 being a deep-water subglacial lake with the dimensions described by Smith *et al*. [27].

The second observation is that Institute E2 does coincide, at least in part, with a small minimum in the subglacial hydrological potential, meaning that basal water is expected to accumulate at some level. The location of this potential minimum is downstream of a subglacial hill. The implication is that Institute E2 is an ephemeral, probably shallow, lake that exists because water pools on the lee side of a subglacial hill; the potential minimum being a result of the ice thickness gradient rather than the basal topography. One possible explanation, given the spatially restricted nature of the hydrological minimum, is that its dimensions are far smaller than those proposed by Smith *et al*. [27].

Clearly, the differences between Institute E2 and Lake Vostok are substantial and they possibly represent end-members of the spectrum of Antarctic subglacial lakes. To see whether other ‘active’ subglacial lakes conform to observations at Institute E2, Wright *et al*. [14] undertook an analysis of airborne RES data across a selection of ‘active’ subglacial lake locations within the Byrd Glacier catchment in East Antarctica, the region where Stearns *et al*. [17] had noticed an association between subglacial lake discharges and ice-sheet flow enhancement. As per Siegert *et al*. [33], no direct evidence for subglacial lakes was found for any of the potential ‘active’ lake sites in the RES data. The RES equipment used was certainly capable of detecting subglacial lakes, as three new lakes were discovered in the same survey (the unique ‘three-tier’ lake system, in which subglacial water cascades up a subglacial hill, pooling as it does so at three locations). Across the whole region, considerable topographic relief was measured, with an abundance of subglacial hills up to 500 m in height obvious in the data. As with Institute E2, it seems likely that the flow of subglacial water in the Byrd Glacier catchment is influenced heavily by subglacial topography and that ephemeral pooling of the water is possible in the lee side of hills. It also seems likely that the horizontal dimensions of ‘active’ lakes derived from satellite observations are far greater than those of the water change responsible. If this is true, for conservation of volume to be upheld, the vertical change in the water level must be greater than the elevation change observed at the ice surface, with the implication being that ‘active’ lakes are relatively deep when filled.

## 3. Ice-shelf channels and evidence of organized subglacial flow of water

**Figure 3.**
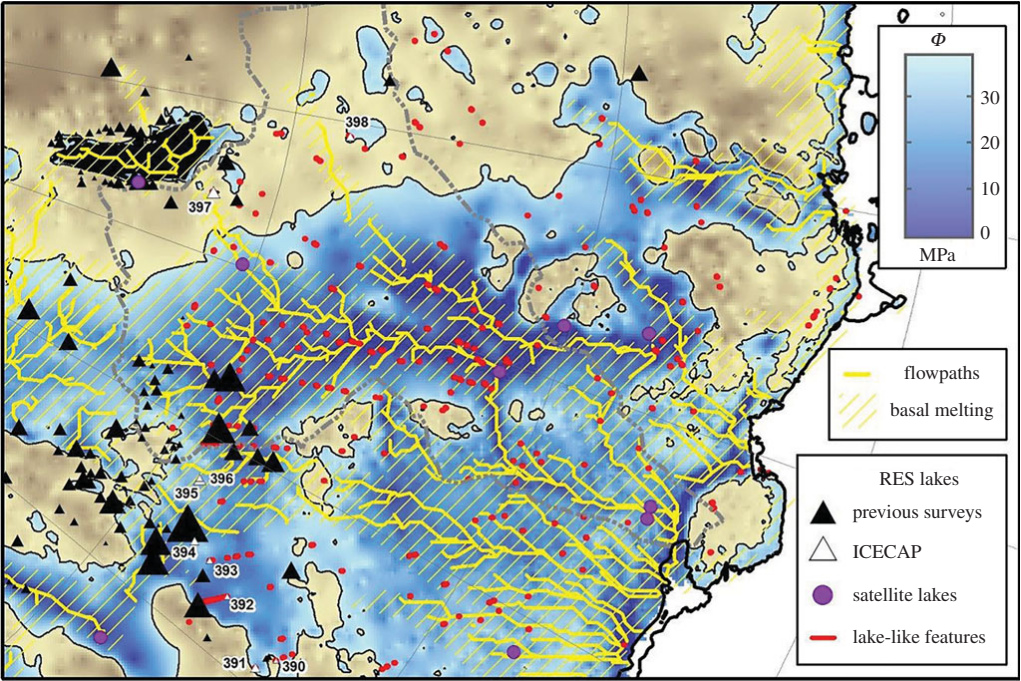
The subglacial hydrology of the Aurora Subglacial Basin area, including the locations of subglacial lakes (triangles) and sites of ice-surface elevation change interpreted as subglacial water movements (purple circles). Sites that do not resemble substantial lakes in RES data, but are identified by an automated algorithm for detecting subglacial water, are also shown as red dots. The extent of the predicted flow paths of subglacial water is limited to areas of subglacial topography at the pressure melting point. Subglacial lakes with numbers relate to new lakes discovered since the last full inventory [12]. (Adapted from Wright *et al*. [13].)

The dynamic behaviour of ‘active’ lakes demonstrates that there are hydrological pathways and connections beneath the ice sheet. Wright *et al*. [13] showed that these connections are capable of transporting subglacial water generated in the centre of the ice sheet to the margins. They used a combination of RES analysis and numerical ice-sheet modelling to reveal that the ice-sheet bed was continuously wet between the subglacial lakes at Dome C in central East Antarctica and the coast at Totten Glacier via the Aurora subglacial basin. As a consequence, they concluded that there was nothing to stop water flowing from the ice-sheet centre to the margin (figure 3). Modelling of ice and water flow in the Siple Coast in West Antarctica also reveals how subglacial water drains through a subglacial lake network and exits at the grounding line [36]. The nature of the basal hydrological system, driven by both periodic lake drainage and a background hydrological flow, is still in question however and this uncertainty has large implications for our appreciation of the interaction between subglacial water and the ice flow above it.

Wingham *et al*. [16] first evaluated the nature of the connections between subglacial lakes, suggesting that an R-channel, incised upwards into the ice, was likely to be a plausible mechanism for draining water issued from ‘active’ lakes. Schroeder *et al*. [37] provide further evidence for channelized water flow through R-channels, from an analysis of the specularity of radio-wave reflections from the ice-sheet base beneath the Thwaites Glacier in West Antarctica (see also [38]). The question of whether the periodic draining of lakes or the background hydrological system are capable of supporting an R-channel-type channelized drainage is still under investigation [39]. Carter *et al*. [40] suggest that it is more likely that the lakes are drained through channels cut into the sediment, for example. Improved inferences of geothermal heat flux [41] suggest that, beneath Thwaites Glacier, it may be more than three times the value used in most ice-sheet models (approx. 200 compared with approx. 60 Wm^−2^). Using the correct (greater) geothermal heat flux will produce higher meltrates than previously calculated, which may be capable of supporting persistent R-channels.

**Figure 4.**
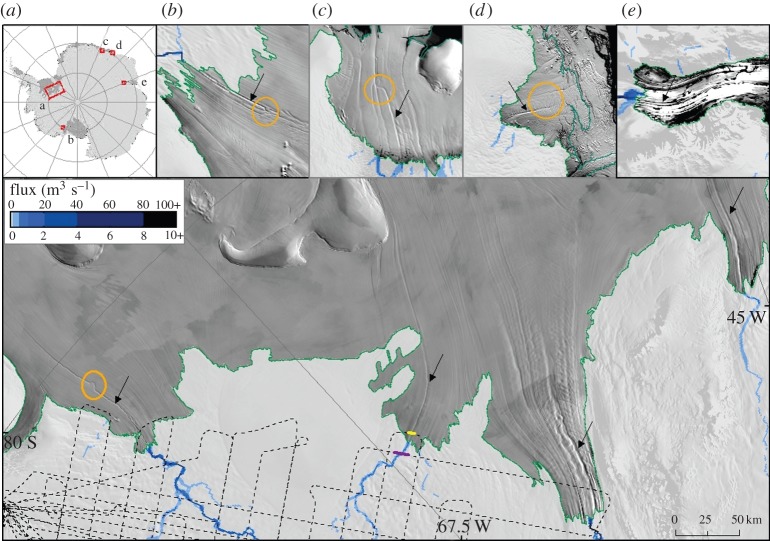
Ice-shelf surface channels visible on the MODIS Mosaic of Antarctica, overlain by calculations of subglacial meltwater flux. (*a*) Filchner–Ronne Ice Shelf; the arrowed features are downstream of (left to right) Institute, Moller, Foundation and Support Force Ice Streams. (*b*) MacAyeal Ice Stream, flowing into the Ross Ice Shelf. (*c*–*d*) A series of small East Antarctic ice shelves. (*e*) Lambert Glacier, which flows into the Amery Ice Shelf. Black arrowshighlight ice-shelf surface channel features. Orange circles indicate evidence of migration of the exit point of subglacial channels. The green line is the MODIS-derived ice-sheet grounding line. Dashed lines on (*a*) are airborne RES flightlines. (Adapted from Le Brocq *et al*. [42].)

Le Brocq *et al.* [42] provide further evidence for water flowing in focused units beneath the ice sheet, though they draw no conclusions about whether the channels are incised into the ice or the sediment. Observations of often sinuous channels on the surface of ice shelves indicate the presence of channels incised upwards into the underside of the floating ice shelf (figure 4). These sub-ice-shelf channels line up with flow routes predicted to be taken by subglacial water drainage, indicating that the ice-shelf channels are cut by meltwater plumes generated by buoyant subglacial water exiting the grounding line in a focused manner. It is not clear whether the background hydrological system is causing these features, or whether lake drainage leads to elevated volumes of meltwater that are capable of forming a temporarily organized drainage system. In some cases, the ice-shelf channels show a level of sinuosity that is only explicable through lateral migration of the basal water exiting the ice-sheet base, suggesting changes to the grounding line position and/or temporal and spatial modifications to the subglacial hydrology system upstream (figure 4; [42]).

## 4. Channels at the bed of the ice sheet

Geomorphological evidence for channels associated with former subglacial flow of water and its connectivity is well documented from the areas of former Northern Hemisphere glaciation (e.g. [43]), from the Antarctic Dry Valleys [44] and from the offshore Antarctic continental shelf (e.g. [45]). We have limited comparable evidence beneath the present-day Antarctic Ice Sheet, however.

**Figure 5.**
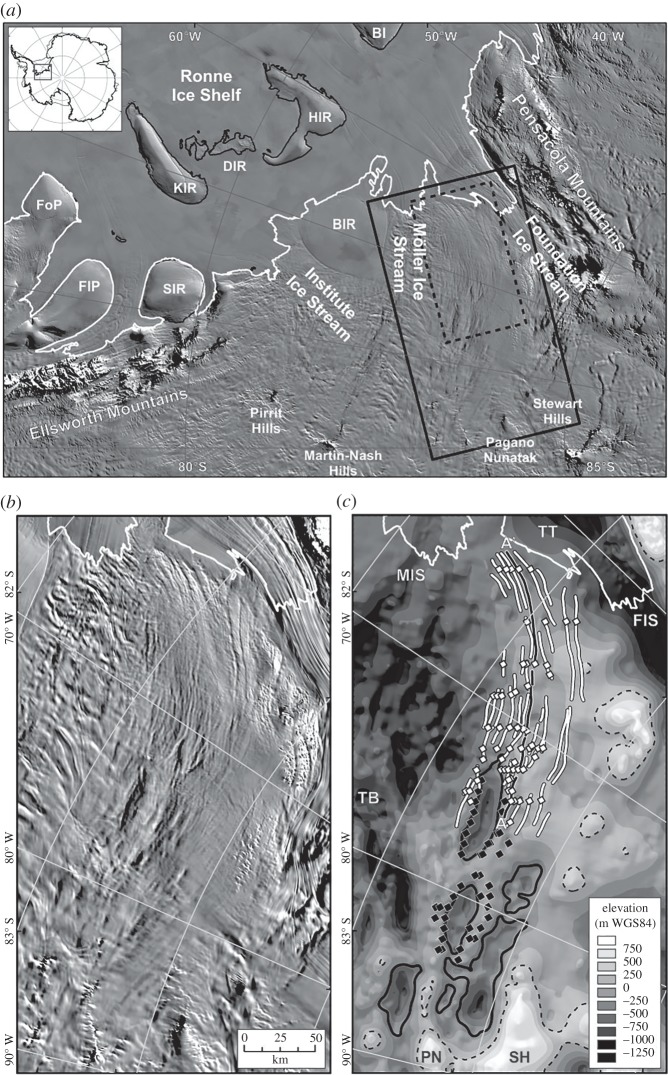
(*a*) MODIS satellite imagery [46] revealing the location of the inner Weddell Sea Embayment, West Antarctica. Black box marks the extent of panels (*b*,*c*). (*b*) Linear surface features identified in MODIS imagery. (*c*) Subglacial topography overlain with channel locations (white lines) observed in (*b*). White diamonds identify subglacial channels that are visible in both the MODIS imagery and in RES data. Black diamonds mark channels only visible in RES data. The black dashed line is the 0 m elevation contour. Solid black lines denote the Marginal Basins (−650 m contour). Annotations are provided as follows: BI, Berkner Island; BIR, Bungenstock Ice Rise; DIR, Doake Ice Rumples; FlP, Fletcher Promontory; FoP, Fowler Peninsula; FIS, Foundation Ice Stream; HIR, Henry Ice Rise; KIR, Korff Ice Rise; MIS, Möller Ice Stream; PN, Pagano Nunatak; SH, Stewart Hills; SIR, Skytrain Ice Rise; TB, Transitional Basins; TT, Thiel Trough (southern edge). (Adapted from Rose *et al*. [47].)

RES data from the Weddell Sea Embayment in East Antarctica reveal a 100 km long, 70 km wide system of major incised channels (5 km wide and more than 200 m deep) and smaller scale canyon-like structures (250 m wide and less than 40 m deep; figure 5) [47]. Equivalent geomorphic features found in the Wilkes Basin, in East Antarctica, were interpreted as subglacial meltwater channels formed during the drainage of a large (850 km^3^) palaeo-subglacial lake in the upper Wilkes Basin in the Middle Miocene [48]. Characterization of such landforms is often restricted by the line spacing of RES surveys, preventing continuous along-track identification and measurement. Such limitations can be overcome if satellite-derived ice-surface imagery and radio-wave backscatter data are available to infer bed properties between RES data. Rose *et al*. [47] used this approach to reveal the presence of a system of ancient large sub-parallel subglacial channels beneath a slow flowing sector of the West Antarctic Ice Sheet between the Foundation and Möller Ice Streams. These subglacial channels (average width 2.6 km, average depth 158.9 m) extend for at least 250 km. They are thought to be caused by flow of water at the bed of the ice sheet, rather than by surface (i.e. fluvial) water flow, as they lie below present-day sea level (average elevation of −623 m) and have undulating longitudinal profiles. The present-day ice sheet over them is cold based, meaning they are inactive as water conduits at present and slow flowing (i.e. less than 5 ma^−1^), meaning the channels have clear surface expressions (i.e. each channel is associated with a narrow elongated depression on the ice-sheet surface, which is apparent in optical and radar mosaics, e.g. [49]). Combining the ice-surface imagery with RES observations, Rose *et al*. [47] were able to map the geomorphic form and extent of the channels in detail. The direction of the channels, at an angle to the maximum ice-sheet surface slope, indicates that, even if the ice sheet were warm based, water would not be routed along them. This implies that a former ice sheet, with different configuration from that of today, was responsible for their formation. The channels are not thought to be the result of the rapid drainage of a former subglacial lake, due to the lack of a potential site upstream for such a lake. Instead, Rose *et al*. [47] interpreted their formation as resulting from the transfer of surface meltwaters to the ice-sheet bed, during a time when the West Antarctic Ice Sheet was temperate and subject to significant amounts of seasonal surface melt. Such meltwater may have acted to fill the Marginal Basins [47], which are located upstream of the channels, and which may have acted to focus the meltwater from upstream. Rose *et al*. [47] postulated that the Pliocene was the most recent date at which this could have occurred, suggesting a highly dynamic West Antarctic Ice Sheet at this time.

While channels at the ice-sheet bed have only been identified across a few locations, it is entirely possible that other regions of the ice-sheet base contain both relic and ‘active’ subglacial drainage features. By combining satellite and airborne geophysical datasets, using techniques established by Rose *et al*. [47] and Jordan *et al*. [48], the importance of extensive connected subglacial drainage systems to the Antarctic Ice Sheet through geological time and their long-term impact as agents of landscape evolution may be evaluated more fully.

## 5. Summary and future work

Research undertaken in the last 4 years has expanded our knowledge of subglacial hydrology considerably and has also allowed a number of new research questions to be framed. Wright & Siegert [12] and Wright *et al*. [13,14] list evidence for 402 Antarctic subglacial lakes. Wright & Siegert [12] also describe the time frame over which lake discoveries have been published, demonstrating an apparent exponential growth in lake discoveries with time. Clearly, the number of actual subglacial lakes is finite, hence the curve must flatten at some stage. If we consider that very few subglacial lakes have been discovered in the past few years, this transition in the time series of subglacial lake identifications may have already started.

The notion of ‘active’ subglacial lakes has provoked speculation that subglacial lake water at the centre of the ice sheet is able to flow to the margin. While direct evidence for such flow will obviously be difficult to acquire, Wright *et al*. [13] have shown that there is little impediment for it at least in the Aurora basin and Totten Glacier region of East Antarctica. Evidence for subglacial water emerging at the margin is compelling [42] and, while there is debate on whether such water derives from subglacial lake discharge, it must be fed from an upstream source. This water is likely to be composed of a combination of local basal melting and more distant sources, the proportions of which are as yet poorly understood.

Another unknown is the size and nature of the basal water bodies that are responsible for ice-surface elevation changes. Research to date points to much smaller water bodies than originally depicted, but more work is needed to fully appreciate the hydrological processes responsible for the surface observations.

The traditional view of Antarctic subglacial hydrology is to consider the ice-sheet bed as being impermeable. This is almost certainly not the case and there is every possibility that groundwater exists across large portions of the continent. Indeed ice-sheet modelling has pointed to groundwater as being critical to maintaining the flow of ice streams in the Siple Coast [50]. Further, Wadham *et al*. [51] postulated that biogeochemical processes within deep water-saturated sediments may have led to substantial stores of methane within a microbially active wetland system beneath the ice. Despite the likelihood of its existence and its potential significance, Antarctic groundwater has never been detected, apart from at the edges of the continent, such as in the Dry Valleys, where highly concentrated brine has been detected using airborne electromagnetic methods [52]. The reason groundwater has yet to be detected is that the geophysical experiment needed has yet to be designed. Indeed, sounding beneath the ice-bed interface, which itself is beneath several kilometres of ice, is conceptually challenging. The solution will probably lie in a combination of geophysical techniques, such as three-dimensional seismics, RES, electro-seismics and electro-magnetics, acquiring data simultaneously at small scale and in high resolution. Several sites where deep basal sediments exist are known (e.g. the Aurora Basin, Siple Coast and Institute Ice Stream). Future targeted geophysical research here may lead to the discovery of a further element at the scientific frontier of Antarctic subglacial research.
